# Analysis of particles containing alpha-emitters in stagnant water at torus room of Fukushima Dai-ichi Nuclear Power Station’s Unit 2 reactor

**DOI:** 10.1038/s41598-022-11334-1

**Published:** 2022-05-16

**Authors:** Takumi Yomogida, Kazuki Ouchi, Toshitaka Oka, Yoshihiro Kitatsuji, Yoshikazu Koma, Katuhiro Konno

**Affiliations:** 1 Nuclear Science and Engineering Center, Japan Atomic Energy Agency, Ibaraki, 319-1195 Japan; 2 Collaborative Laboratories for Advanced Decommissioning Science (CLADS), Japan Atomic Energy Agency, Ibaraki, 319-1194 Japan; 3 Fukushima Daiichi Decontamination and Decommissioning Engineering Company, Tokyo Electric Power Company Holdings Inc., Fukushima, 979-1301 Japan

**Keywords:** Environmental chemistry, Nuclear fuel

## Abstract

Particles containing alpha (α) nuclides were identified from sediment in stagnant water in the torus room of the Fukushima Dai-ichi Nuclear Power Station(FDiNPS)’s Unit 2 reactor. We analyzed uranium (U), which is the main component of nuclear fuel, using scanning electron microscopy (SEM). Other α-nuclides (plutonium [Pu], americium [Am], and curium [Cm]) were detected by alpha track detection and the morphology of particles with α-nuclides were analyzed by SEM-energy dispersive X-Ray (EDX) analysis. Several uranium-bearing particles ranging from sub-µm to several µm in size were identified by SEM observation. These particles contained zirconium (Zr) and other elements which constituted fuel cladding and structural materials. The ^235^U/^238^U isotope ratio in the solid fractions that included U particles was consistent with what was found for the nuclear fuel in the Unit 2 reactor. This indicated that the U of similar fuel composition had made finer. The α-nuclide-containing particles identified by alpha track analysis were several tens to several hundred µm in size. The EDX spectra showed that these particles mainly comprised iron (Fe). Since the amount of α-nuclide material was very small, Pu, Am, and Cm were adsorbed on the Fe particles. This study clarifies that the major morphologies of U and other α-nuclides in the sediment of stagnant water in the torus room of FDiNPS’s Unit 2 reactor differed.

## Introduction

TEPCO's Fukushima Dai-ichi Nuclear Power Station (FDiNPS) was severely damaged by the earthquake and resulting tsunami that struck on March 11, 2011^1^. At the time, Units 1–3 of the six reactors were in operation, and the nuclear fuel in the Units 1–3 reactors was damaged. Seawater and freshwater were injected to remove the decay heat from the nuclear fuels. The water remained in the basement of the building, and the components of the nuclear fuel dissolved in it, resulting in highly radioactive stagnant water. The stagnant water contained radionuclides, such as fission products and actinides derived from nuclear fuels. A chemical treatment process was established to remove the radionuclides, and a recirculating engineering system was established to reuse the recovered cooling water. Since then, the amount of stagnant water has been gradually reduced, but it was discovered that the fine particles containing a higher concentration of Alpha (α)-emitting radionuclides were settling basement in the reactor building^2^. The concentrations of alpha-nuclides (10^2^–10^5^ Bq/L) in the stagnant water including sediments were higher than the cooling water in the downstream building. Alpha-emitting radionuclides such as uranium (U) and plutonium (Pu) can cause serious internal exposure upon entering the human body. Alpha-nuclides should be strictly controlled when compared to caesium(Cs)-137 and strontium(Sr)-90, which are the main nuclides in fission products. Technology must be developed to effectively remove the alpha-nuclides from the stagnant water. For this purpose, we collected stagnant water in the torus room in the basement of the reactor building of Unit 2 and conducted radiochemical analysis of the precipitates in the stagnant water.The stagnant water is a higher concentration compared with what was detected at the entrance to the Cs adsorption system. In addition, the presence of α-emitting radionuclides was confirmed in the samples containing mixed sludge components from the stagnant water in the reactor building. To proceed with the treatment of the stagnant water deep inside the reactor building in the future, a better understanding is required of the different types of α-emitters, particularly those included in particulate solids in the stagnant water.

In existing research, radioactive particles containing U were detected in association with Cs microparticles (CsMPs) outside the FDiNPS site and their physicochemical composition and morphology were analyzed^3–8^. Abe et al.^3^ collected CsMPs emitted from the FDiNPS from the atmosphere and analyzed them using synchrotron radiation X-rays to detect U in the CsMPs. Ochiai et al. detected U particles of several hundred nm in CsMPs by scanning electron microscopy-X-ray detection (SEM-EDX) analysis. Their results reflected the composition of UO_2_ on magnetite by observing the diffraction pattern obtained using transmission electron microscopy. Similarly, diffraction patterns of UO_2_ and zirconia were obtained from mixed particles of Zirconium (Zr) and U in CsMPs, respectively. This indicated that U was present in CsMPs in both UO_2_ nanocrystals and U-Zr nanocrystrals forms^6^. Kurihara et al.^8^ found that the U in the fuel composition of the Unit 2 reactor was present in the CsMPs by analyzing the isotope ratios of ^235^U and ^238^U in the CsMPs using nanoscale secondary ion mass spectrometry. The release of fuel-derived Pu into the environment has also been reported by soil analysis^9–13^, airborne particles^14^, and CsMPs^7^. For americium (Am) and curium (Cm), few reports have been published regarding their release into the environment^11^. Recently, Morishita et al.^15^ detected particles containing α-emitters in smear samples collected from inside the FDiNPS using an α-ray imaging detector. The maximum energy of the α-rays indicated the presence of ^238^Pu; γ-ray spectra indicated the presence of ^241^Am. The morphology of these α-emitters was not observed.

In this study, we analyzed the concentrations and forms of U and other α-emitters in liquid and solid phases to obtain the basic data necessary for considering a removal method for α-emitters in the stagnant water of Unit 2 of the FDiNPS. The search for radioactive particles in existing studies was conducted primarily using imaging plate (IP)^4^ or sodium iodide scintillation counters^8,16^ and by detecting γ-rays from CsMPs. However, while these methods are effective for CsMPs with high radioactivity, it is difficult to selectively detect α-emitters that are present in small amounts and with low specific radioactivity. Therefore, we decided to use a combination of an automated particle measurement method using SEM-EDX^17^ and a method for detecting particles containing α-emitters using solid-state track detectors^18–24^.

## Results and discussion

### Particle size distribution of solids in the stagnant water containing uranium and alpha-emitters

**Figure 1:**
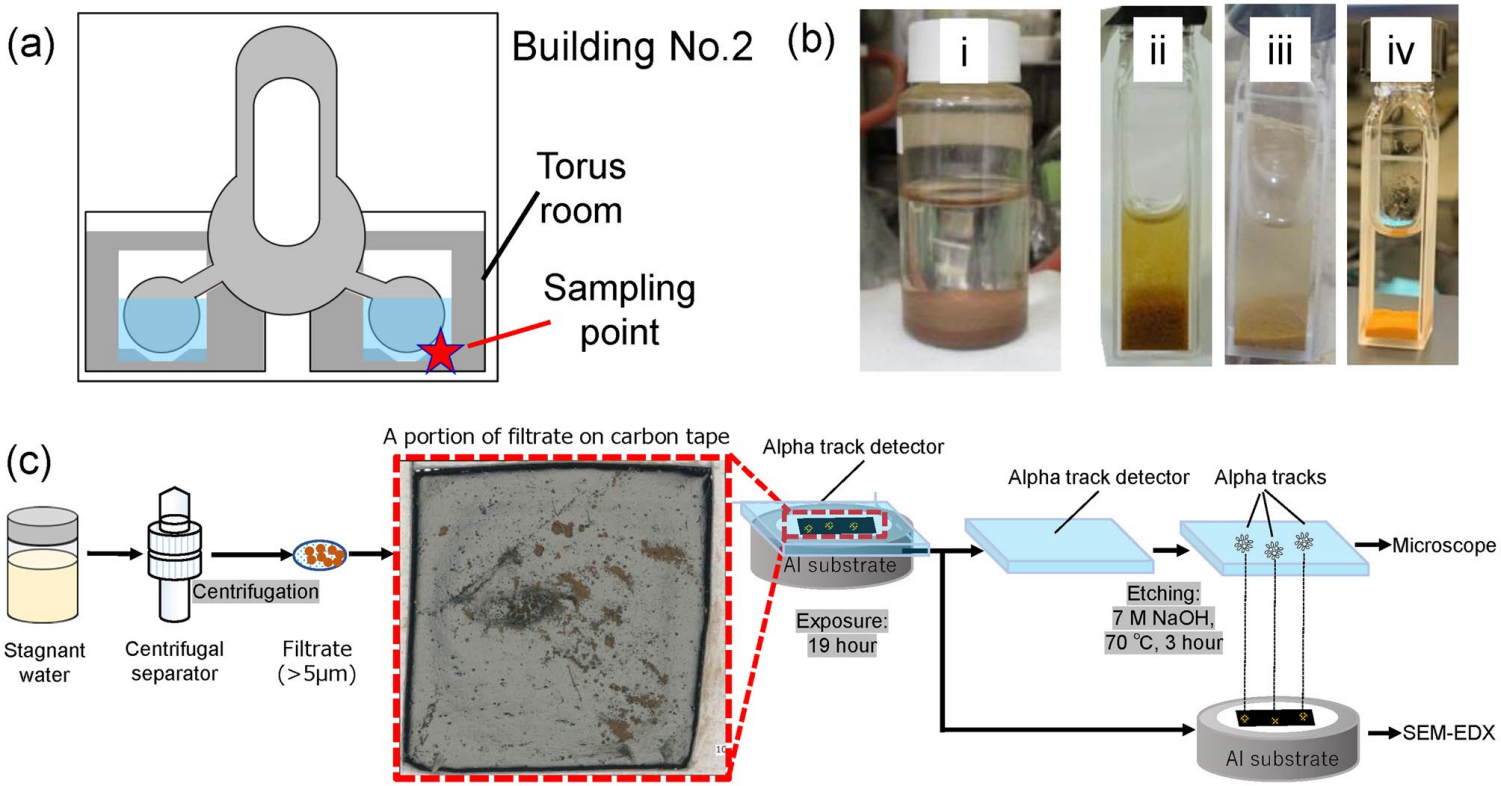
(**a**) Schematic of sampling location. (**b**) Photographs of stagnant water: (i) original solution and (ii) 0 min, (iii) 20 min, and (iv) 2 h after separation from the original solution. (**c**) Schematic of sample preparation procedure for alpha track detection and SEM–EDX analysis.

Figure 1a shows a schematic of a sampling location of a stagnant water sample in this study. Figure 1b shows how the particles settled after the sample was collected. The reddish-brown particles had settled over time. The solids in the stagnant water were classified and the U concentration of each fraction was measured by inductively coupled plasma mass spectrometry. The results are shown in Table 1.

**Table 1:** U concentrations in the fractions of the stagnant water (SW) sample.

	SW-1		SW-2		Average			Difference from U-235/U-238 of Unit 2*^3^(%)
	U-235 (ppb)	U-238 (ppb)	U-235 (ppb)	U-238 (ppb)	U-235 (ppb)	U-238 (ppb)	U-235/U-238	
Residues on 10 µm filter	7.94	4.35 × 10^2^	9.29	4.78 × 10^2^	8.62	4.57 × 10^2^	1.89 × 10^–2^	−1.1
Residues on 1 µm filter	< 7.41 × 10^–3^	1.36 × 10^–1^	< 7.41 × 10^–3^	1.42 × 10^–1^	< 7.41 × 10^–3^	1.38 × 10^–1^	–	–
Residues on 0.1 µm filter	< 7.47 × 10^–3^	1.84 × 10^–1^	< 7.47 × 10^–3^	1.51 × 10^–1^	< 7.47 × 10^–3^	1.68 × 10^–1^	–	–
Residues on 0.02 µm filter*^1^	6.12 × 10^–2^	3.01	1.23 × 10^–2^	5.26 × 10^–1^	3.68 × 10^–2^	1.77	2.08 × 10^–2^	0.9
Filtrate of 0.02 µm filter	< 1.01 × 10^–2^	2.28 × 10^–1^	< 1.01 × 10^–2^	1.63 × 10^–1^	< 1.01 × 10^–2^	1.95 × 10^–1^	–	–
Total amount*^2^	8.00	4.39 × 10^2^	9.30	4.79 × 10^2^	8.65	4.59 × 10^2^	1.89 × 10^–2^	−1.2

*1 Difference in U concentration between a 0.1 and 0.02-µm filter filtrate.*2 Sum of the U concentrations of the 10-, 1-, and 0.1-µm filter residues and the 0.1-µm filter filtrate.*3 Isotope ratio of U-235/U-238 in Unit 2 was 1.93 × 10^–2^ according to the JAEA Data/Code 2012–018 p18.

As indicated, ^238^U was quantified in all fractions of all particle sizes, indicating its existence in various particle sizes. More than 99 % of U was present in fractions larger than 10 μm. The ^235^U/^238^U isotopic ratio was approximately 1.9 %, which closely matched the Unit 2 composition (1.93 %)^25^. Analysis of the total α-activity in each fraction showed that more than 99.8 % of the α-emitters were present in fractions larger than 10 μm (see Supporting Information, Table S1). These results suggested that most of the U and α-emitters in the stagnant water sample of the Unit 2 were present in particle fractions larger than 10 μm. Accordingly, a search for particles containing U and α-emitters was attempted using particles in solid fractions.

### Detection and composition analysis of uranium particles using scanning electron microscopy-X-ray detection

**Figure 2:**
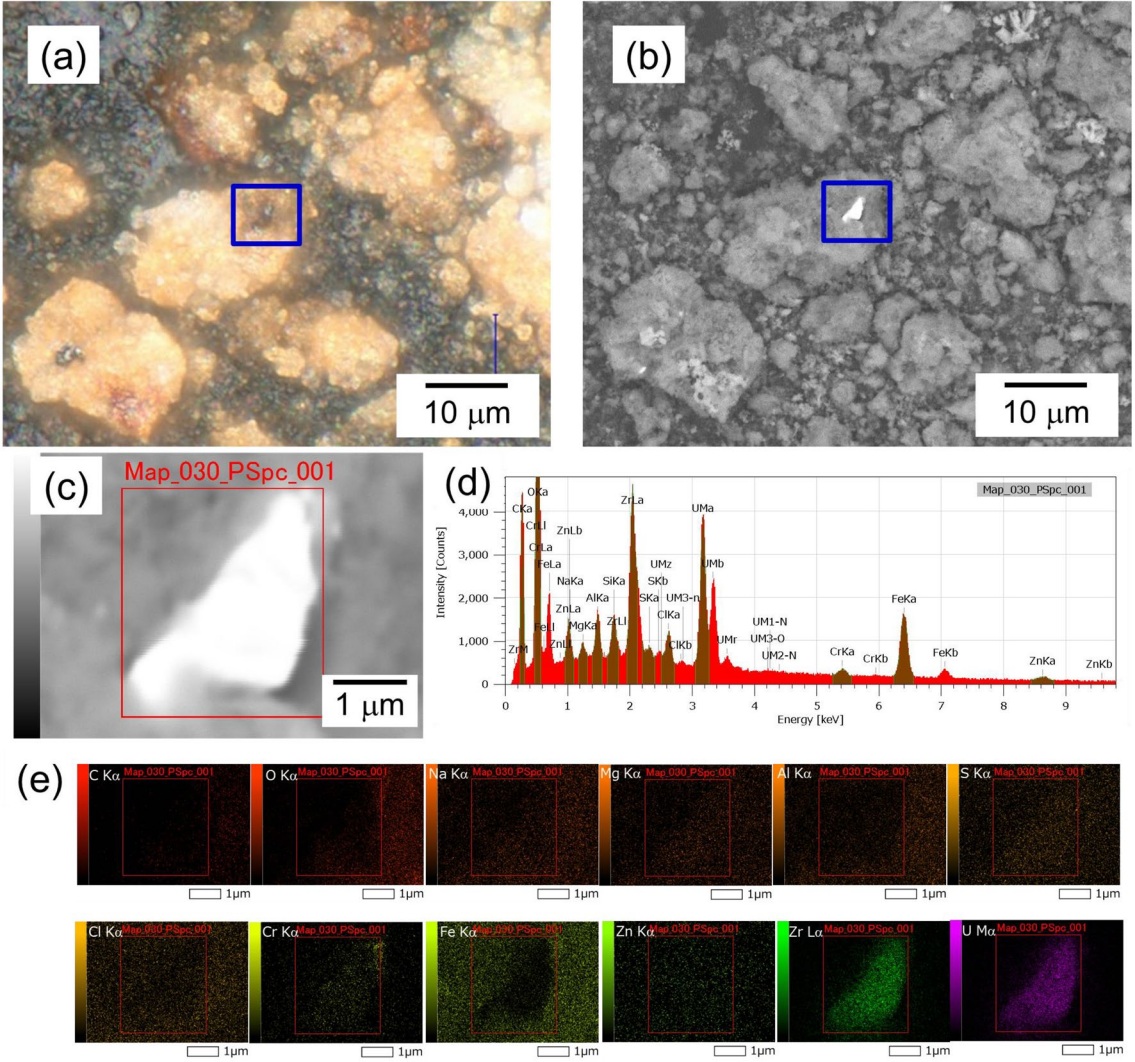
Example of the detection of U particles using SEM–EDX. (**a**) Optical image and (**b**) back-scattered electron detection (BED) image of U particle (UP1) on Fe particles. (**c**) The BED image at high magnification indicated by the blue square in (**b**). (**d**) EDS spectrum of the area indicated by the red square in (**a**). (**e**) Elemental maps of the major content in the U particles.

As the main U isotopes (^235^U and ^238^U) in the fuel composition have a long half-life and low specific activity, SEM-EDX was adopted to detect U-rich particles. Precipitates on the filter with a pore size of 5 μm were loaded onto carbon tape (Fig. 1c) and observed by SEM-EDX. Particles containing more than 3 % U by atomic ratio (hereafter referred to as “U particles”) were detected based on the results of elemental composition analysis. An example of the observation result of UP1 is shown in Fig. 2.

Black particle (UP1) was observed on top of the reddish-brown particles in the center of optical image in Fig. 2a. Figure 2b shows a backscattered electron detection(BED) image of the same region as Fig. 2a. In the BED image and its magnified view (Fig. 2b, c), the black particle in Fig. 2a had a high intensity. In general, a particle containing an element with a relatively higher atomic number yields a BED image with higher brightness. The particle (UP1) with high brightness in Fig. 2b, c should contain heavy element. The peaks at 3.18 keV (U Mα), 3.34 keV (U Mβ), and 3.55 keV (U Mγ)^26^ were observed within the EDX spectrum of the UP1 particle (Fig. 2d), indicating that the particle included U. According to the results of SEM-EDX composition analysis of the U particles (UP1 in Table S2), U was the main component. In addition, the distribution of components in the fuel-structure materials, such as Zr and Cr, was also observed on the U particle (Fig. 2e). In contrast, iron (Fe) was observed to have been distributed around the U particles, indicating that the U particles were attached to the Fe particles. These results suggested that the U particle would be particulate with fuel-structural materials.

**Figure 3:**
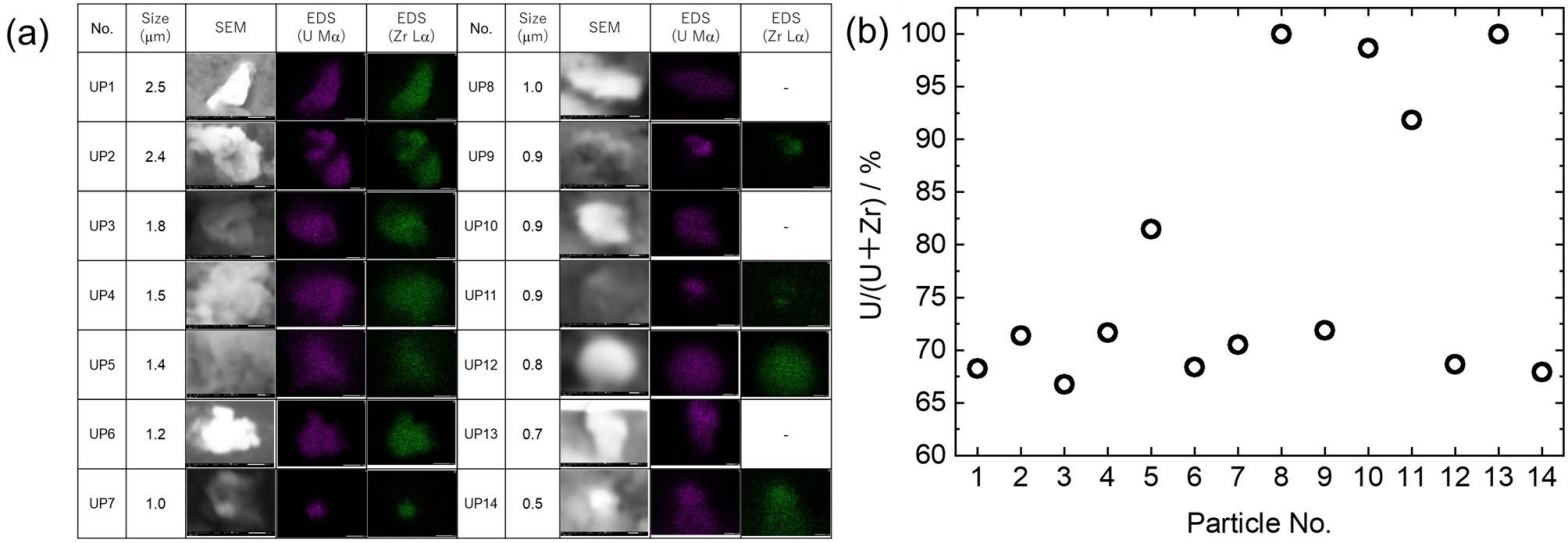
U particles that were obtained from the residue on the polycarbonate filter with a pore size of 5-μm diameter. (**a**) SEM images and elemental maps of the U particles. (**b**) Molar ratios of U/(U + Zr), which were analyzed using SEM–EDX.

Using the same procedure as in the above paragraph, 14 U particles were detected. The observed particle sizes and the elemental maps of U and Zr are shown in Fig. 3a. Elemental composition of U particles are shown in Table S2.

Uranium particles with size ranging from approximately 500 nm to 3 μm were observed. Uranium particles were attached to Fe particles or present on their own (Fig. S1). The content of Fe in the analyzed stagnant water sample was approximately 4,400 times higher compared with U, indicating that the main component of the filtered material was Fe. Uranium particles smaller than the filter pore diameter of 5 μm were trapped, suggesting that they were cake-filtered during centrifugal filtration.

The isotope ratios of U in this fraction were consistent with the fuel composition derived by ICP-MS measurements. The presence of U particles with an isotopic composition the same as the nuclear fuel suggested that these U in the stagnant water sample had been derived from reactor core. The release of U and Pu from the FDiNPS into the environment was investigated and clarified by analyzing bulk soil samples for Pu^9–13^ and measuring the isotopic composition of U^3,6,9^ and Pu particles^7^ associated with CsMPs. Fine U, U, and Zr particles, ranging in size from several tens of nm to several hundreds of nm, have been detected in association with CsMPs in the environment^6^. In the present study, it was found that particles approximately 10 times larger in size than the particles associated with CsMPs existed in the stagnant water sample. It was also clarified that some U particles were not associated with CsMPs but existed independently.

Many U particles included Zr, which would have been derived from cladding. The ratios of U and Zr in these particles are compared in Fig. 3b. The ratio of U to Zr in each particle varied. In addition, in some particles, Zr was not detected, suggesting that the particles retained their fuel form. An existing report^6^ suggested the existence of two types of U particles several hundred nm in size that had been derived from the FDiNPS and released into the environment; one of these particles was in the fuel form of UO_2_ and the other presented as a Zr mixed oxide.

### Detection and analysis of particles containing alpha-emitters using alpha track detection

**Figure 4:**
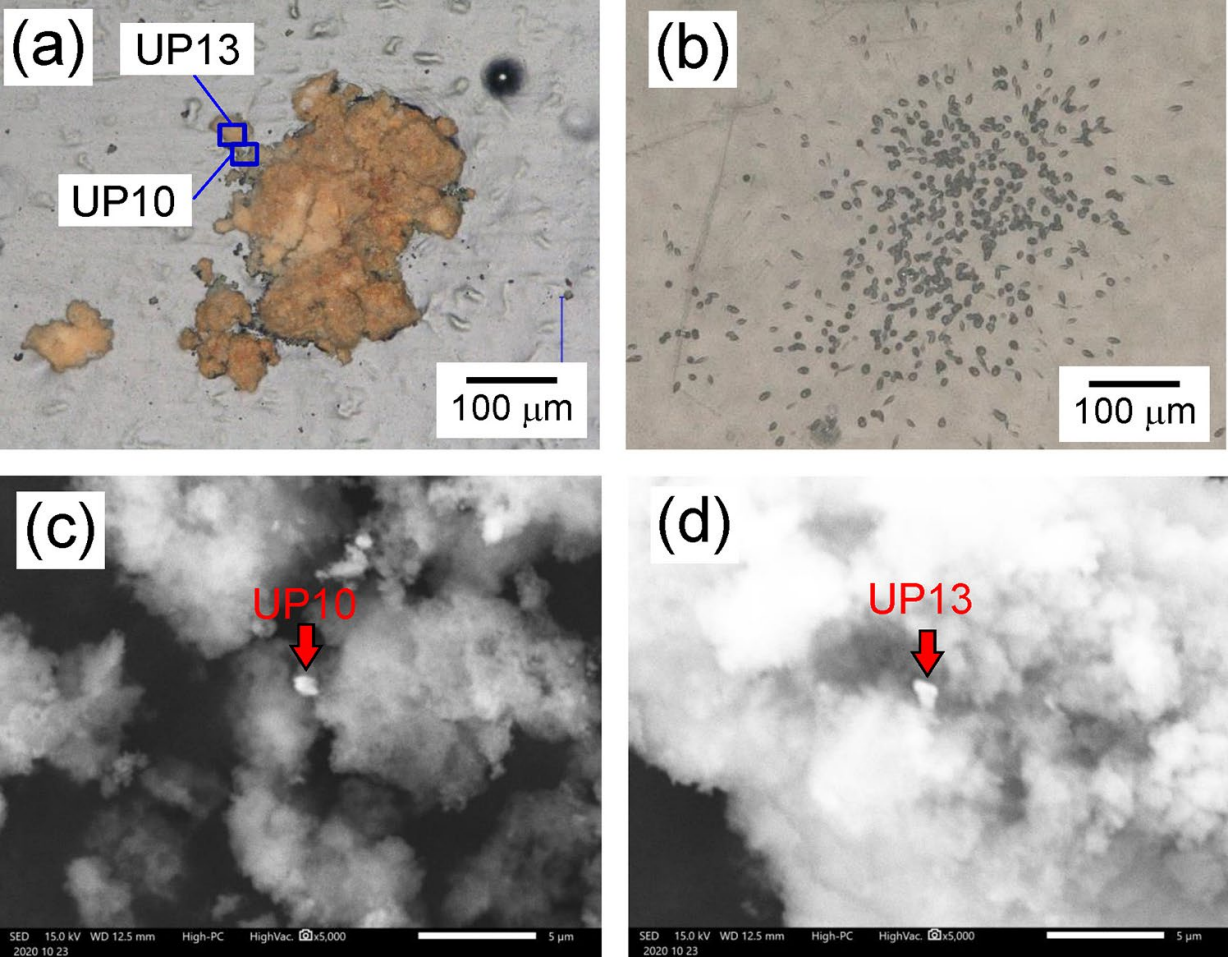
Example of an α-emitter particle. (**a**) Optical image of typical α-emitter particles, i.e., Fe particles with U particles. (**b**) Alpha tracks of the particles in (**a**). (**c**) Magnified SEM images for UP10 and (**d**) UP13 as indicated by the blue square in panel (**a**).

The distribution of α-emitters in solids was investigated using alpha track analysis. An example of α-emitter particles and observed alpha tracks is shown in Fig. 4a, b. The upper left part of the particle in Fig. 4a shows the presence of U particles UP10 and UP13 (Fig. 4c, d), which are identical to those shown in Fig. 3. The distribution of alpha tracks can be observed uniformly from the reddish-brown particles; the uneven distribution of the position of U particles is not presented. Only a few tens of alpha tracks were observed, even for the alpha tracks at the position of the UP1 particle (Fig. S2), which had the largest particle size among the detected U particles with a diameter of 3 μm (Fig. 3a). In comparison, several hundred alpha tracks can be observed in Fig. 4b. This result suggested that the main source of alpha tracks was not U particles but other α-emitters on the reddish-brown particles.

**Figure 5:**
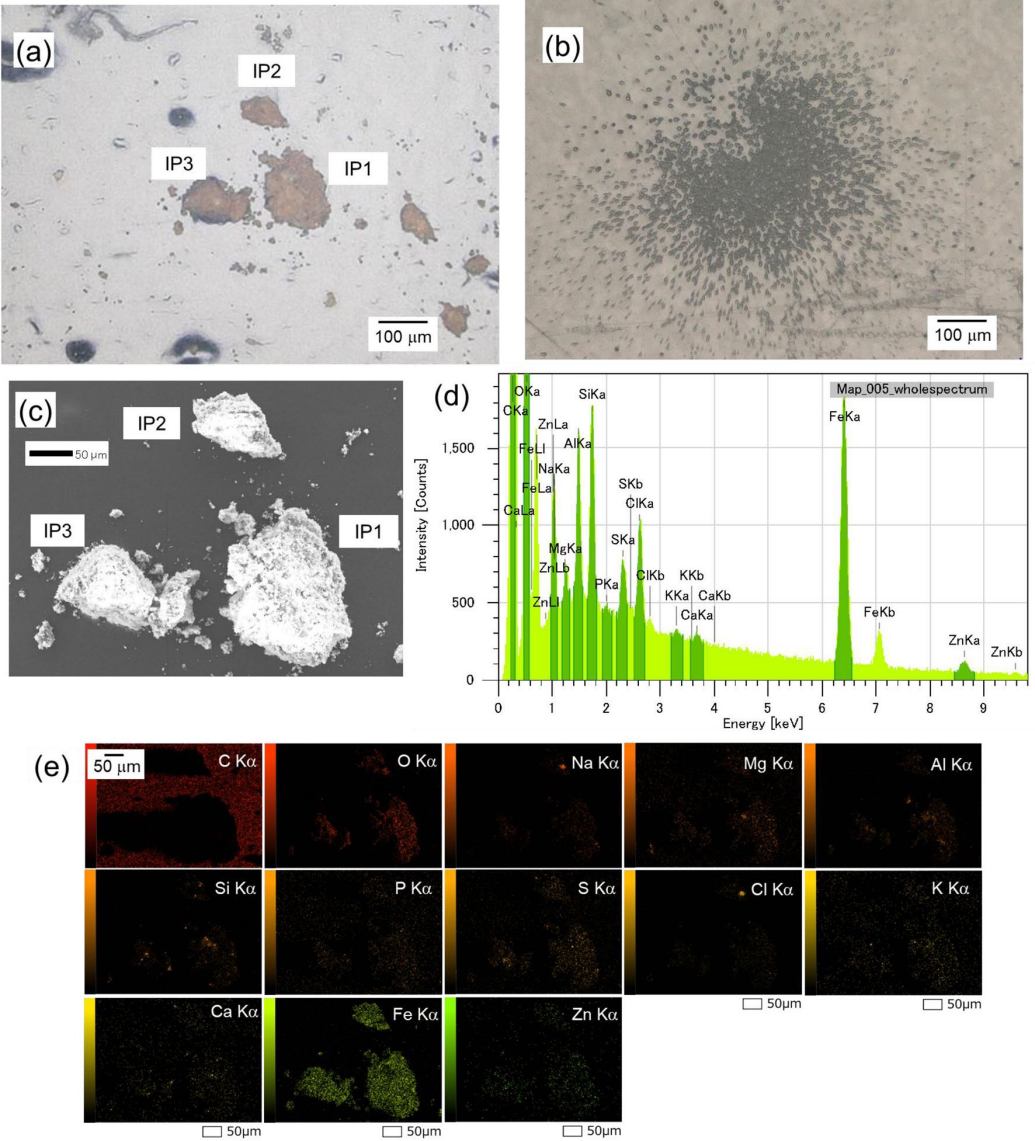
Typical α-emitter particles. (**a**) Fe particles with the most detected alpha tracks. (**b**) Alpha tracks of the particles in (**a**). (**c**) SEM image of Fe particles. (**d**) EDS spectrum of the area in (**a**). (**e**) Elemental maps showing the major contents of the Fe particles.

The particle with the most alpha tracks is shown in Fig. 5a, and the alpha tracks derived from this particle are shown in Fig. 5b. The SEM-EDX observation of this particle is shown in Fig. 5c. Three particles can be observed in this image, all of which were found to comprise mainly Fe, based on the elemental mapping results (Fig. 5d, e). Furthermore, the elemental analysis results showed that U and other α-emitters were not detected (Fig. 5d). An almost uniform distribution was observed of α-nuclides on the Fe particles; this indicated that the α-nuclides present in ionic form in the solution may have focused on the Fe particles.

Uranium was also detected in the fractions smaller than 10 μm; however, α-emitters were only detected in fractions larger than 10 μm (Table S1). As Fe particles were in much larger quantities and sizes than U, the most of Fe particles were present in fractions larger than 10 μm by cake-filtration. As a result, most of the α-emitters, such as Pu, Am, and Cm, were observed the fraction lager than 10 μm, although fine U particles were present in each fraction. Iron particles (Fig. 5) were approximately 100 μm in size, which were smaller than those in Fig. 4 (approximately 200 μm). The number of alpha tracks was much larger among the Fe particles in Fig. 5 than in Fig. 4. Thus, the amounts of α-nuclides were not proportional to the size of the Fe particles but varied between particles. As noted above, the contribution of U particles to the alpha tracks was minimal (Fig. S2), and the number of other α-emitters in U particles was negligible compared with the total amount of α-emitters.

**Figure 6:**
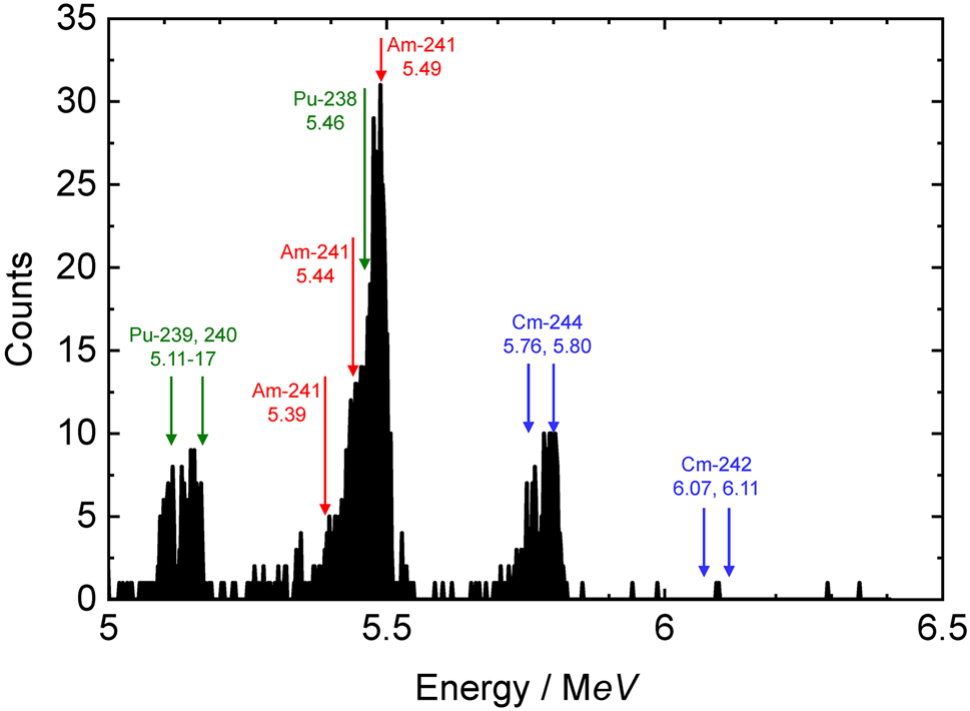
Alpha spectrum of the IP1.

Among the three particles, the IP1 particle, i.e., the main source of the alpha tracks, had been dissolved and α-ray measurements were performed. Figure 6 shows the α-ray spectra of the IP1. Alpha nuclides such as ^239^Pu, ^240^Pu, ^241^Am, and ^244^Cm were detected. For the particle analyzed in this study (IP1), the radioactivity ratio of ^238^Pu + ^241^Am to ^239^Pu + ^240^Pu was 4.03, and the ratio of ^244^Cm to ^239^Pu + ^240^Pu was 1.17, which was almost consistent with the fuel composition (4.50 and 1.14, respectively)^25^. Future work should clarify whether the variation of nuclides exists in each Fe particle.

### Estimation of the chemical properties of uranium and alpha-emitter particles using micro-Raman spectroscopy

**Figure 7:**
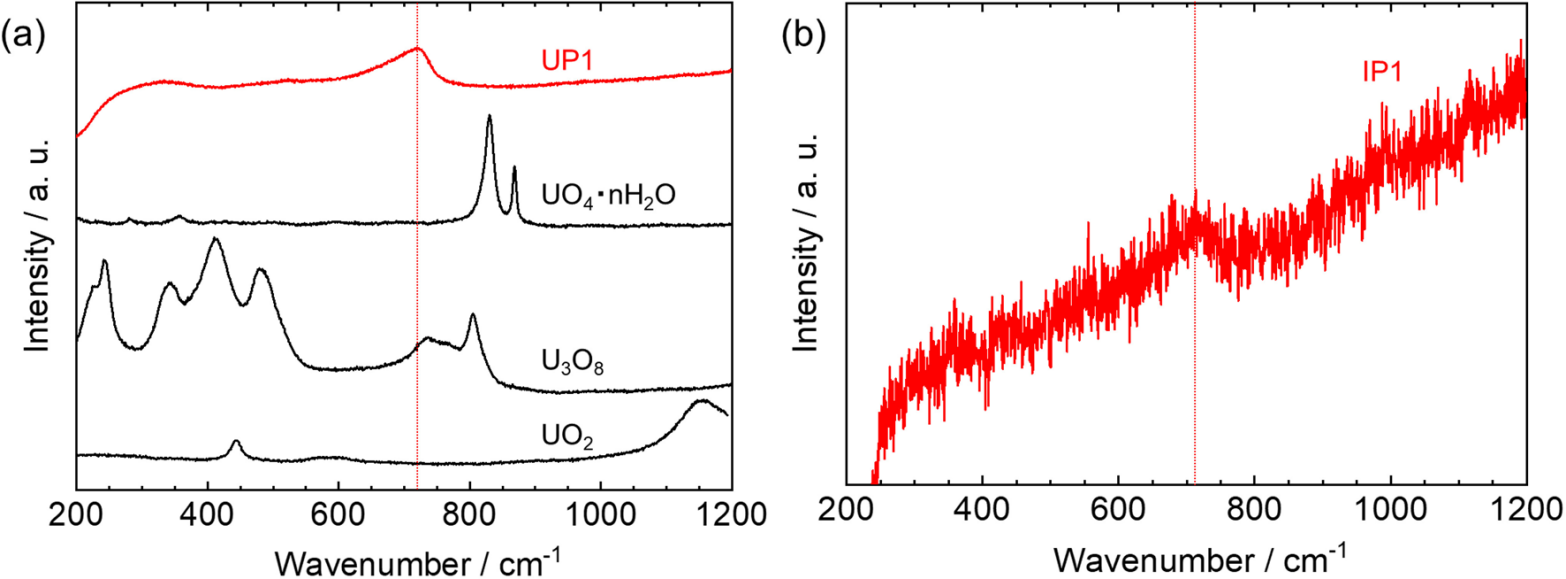
(**a**) Raman spectra of UP1 and uranium standard samples. (**b**) A Raman spectrum of IP1.

A Raman spectrum of the UP1 particle was obtained by micro-Raman spectroscopy. Figure 7a shows the Raman spectra obtained from the UP1 and uranium standard samples. The Raman peak of UP1 is located at approximately 730 cm^−1^, suggesting that it is in a different chemical state from UO_2_ and other U oxides. Figure 7b shows the Raman spectrum obtained from IP1. A Raman peak is only located at approximately 710 cm^−1^. Hanessh reported that natural ferrihydrite has only strong 710 cm^−1^ band^27^.The Raman spectrum of IP1 showed that the surface of the Fe particles existed as ferrihydrite. Since the pH of the stagnant water sample was almost neutral and the adsorption of Pu^28,29^ and Am^29^ on Fe oxides was previously reported, the ions or colloids of these α-emitters would be adsorbed onto the Fe particles. Accordingly, it is suggested that α-nuclides are distributed on Fe oxides.

## Conclusion

To eliminate the presence of α-emitters in the stagnant water, the particles were collected according to their size. Uranium particles were detected by SEM-EDX. Other α-emitters (Pu, Am, and Cm) were detected using alpha track detection and measured via alpha spectrometry. The average isotopic composition of U in the stagnant water sample match well with the fuel composition of FDiNPS’s Unit 2. The U particles in this sample were up to 10 times larger in size than those observed in the environment. It was also shown that Pu, Am, and Cm α-emitters were adsorbed onto Fe particles. These results demonstrated that the major morphology of U and other α-emitters was different. By understanding these types of α-emitters, important information was obtained for considering the separation method of α-emitters in the treatment of the stagnant water in the Unit 2.

## Methods

### Sampling of stagnant water in the torus room of FDiNPS’s Unit 2

A 40-mL of stagnant water sample in torus room of FDiNPS’s Unit 2 was provided form TEPCO HD. The stagnant water containing sediment accumulated on the basement floor was collected with a water sampler at 30th June, 2020.

### Classification of solids and the distribution of uranium and alpha nuclides in the stagnant water

A 2-mL sample of the stagnant water was collected with a stirring well and transferred to a centrifugal ultra-holder (UHP-13C; Advantec) equipped with a 10 µm pore-size membrane filter (PTFE, o.d.13 mm; Merck). This centrifugal ultra-holder was set in a centrifugal separator (CN-820; Az-one) and centrifuged at a rotation speed of 3000 rpm for 10 min to separate the residue from the filtrate. The filtrate was sequentially filtered through 1, 0.1, and 0.02 µm filters. To dissolve the α-emitters in the residue and filtrate, each sample was transferred to a quartz beaker. Nitric acid (HNO_3_) and hydrogen peroxide (H_2_O_2_) solutions were added to the residue and the filtrate on the filter to create a 2 M HNO_3_–2% H_2_O_2_ solution, which was heated and dissolved on a hotplate at 130 °C for 1 h. Since the 0.02 µm pore size of the Anopore membrane filter (0.02 µm pore size; Whatman) was dissolved by HNO_3_ and the impure U contained by the filter was eluted, determination of the residue in the 0.02-µm section was derived from the difference in U concentration in the filtrate of the 0.1 and 0.02 µm filters. The heated sample solution was passed through a UTEVA-Resin column (UT-C20-A; Eichrom) conditioned with 6 mL of 2 M HNO_3_; 15 mL of 2 M HNO_3_ was used to wash out impurities in the column, and 10 mL of 0.01 M HNO_3_ was passed through to elute U adsorbed in the column. The collected eluate was heated on a hotplate at 130 °C until just before it dried up and then re-dissolved in 5 mL of 0.32 M HNO_3_ to make the solution for the ICP-MS measurement. Quantitative analyses of ^235^U and ^238^U were performed by ICP-MS (7700x ICP-MS; Agilent) in the “no-gas” mode using the calibration curve method with a natural U solution. The same procedure was repeated two times (sample name: SW-1,-2).

### Detection of particles containing alpha-emitters using a solid-state nuclear detector

A 1 mL sample of stagnant water was taken and particles were collected by centrifugal filtration using a filter with a pore size of 5 µm (Millipore). Some of the collected particles were transferred to a carbon tape attached to an aluminum sample table using micro spatulas. The sample was placed on top of a solid-state track detector (TNF-1; Hartzlas) and exposed to alpha-rays from the sample for 19 h. Following the exposure, the detector was etched with a 7 M sodium hydroxide solution at 70 °C for 3 h. After the etching process, the detector was ultrasonically cleaned three times using ultrapure water and dried with a clean wipe. The alpha tracks created on the solid-state track detector were observed using an optical microscope (VHX-5000; Keyence), and the location of the particles with high concentrations of α-emitters was identified. The identified α-rich particles were analyzed to observe their composition using SEM-EDX (JEOL, JCM-7000).

Each of the three particles in the spot where the largest number of alpha tracks were observed was transferred onto a 5 mm square silicon (Si) chip using a micromanipulator (QP-3RH; MicroSupport). The micromanipulator was attached to a sampling tool (MTW-1; MicroSupport) and set with micro tweezers (TW-2525; MicroSupport). Under observation using a ×100 to ×1000 objective lens (VH-Z1000R; Keyence) in a microscope, the microparticles were separated using the micro tweezers and placed on the Si chip. The Si chip loaded with the microparticles was transferred to a quartz beaker using ceramic tweezers (TA-CK-20; Toray). Then, 2 mL of 2M HNO_3_ + 2% H_2_O_2_ was added to the beaker and heated on a hotplate at 150 °C for 1 h to dissolve the microparticles, then it was heated on a hotplate at 180 °C for approximately 1 h. Next, The Si chip was cleaned while removing it from the quartz beaker using 5 mL of 0.5 M HNO_3_. The mixture of sample and the rinsing solution was re-dried on a hotplate at 180 °C for approximately 1 h and 30 min. Next, 2 mL of 0.5 M HNO_3_ was added and the sample was heated on a hotplate at 180 °C for approximately 30 min. When the sample solution was approximately 0.1 mL, it was removed from the hotplate. The sample preparation was performed by heating a sample holder for α-ray measurement (o.d.20 mm, stainless steel) at 100 °C, then dropping the sample solution to spread it in the center and baking it on the sample holder.

### Detection and analysis of uranium-containing particles using scanning electron microscopy with energy dispersive X-ray analysis

The same sample that had been used to complete alpha track analysis was used for U-containing particles larger than 0.5 µm in diameter using the automatic particle finder^17^ of the SEM-EDX. First, the field of view was fixed by observing the back-scattered electron image of part of the sample for observation at a magnification of ×1500. Then, in the field of view, the lower limit of brightness was set so that heavy elements beyond Zr could be detected; heavy element particles were automatically detected. The detected particles were automatically elementally analyzed and identified as particles containing more than 3% U by atomic ratio, based on the results of elemental composition analysis. For each U particle detected by the automated particle finder, EDX mapping analysis was performed to determine the elemental composition of U particles. The U and Zr ratios were calculated from the intensity of the 3.18 keV (U Mα) and 2.04 keV (Zr Lα) lines, which were obtained from the EDX spectra of the U particles.

### Microscopic Raman spectroscopic analysis of uranium particles and alpha-emitter particles

The micro-Raman spectrometer (Micro-RAM 532A; Lambda Vision Inc., Japan) used in this study was equipped with a 532-nm neodymium-doped yttrium aluminum garnet laser and a Raman charge-coupled device detector. The laser was focused onto the sample using a ×100 magnification objective lens. The laser power at the sample position was measured using an optical power meter (3664; Hioki Inc., Japan). In this study, the laser power at the sample position was adjusted to 0.4 mW for the measurement of U particles. The acquisition times measured 60 s. Each spectrum made of five accumulations was acquired for each particle. For the measurement of each standard U particle, the laser power at the sample position was adjusted to 0.03 mW. The acquisition times measured 60 s. Each spectrum made of ten accumulations was acquired for each uranium particles. For the measurement of α-emitter particles, the laser power at the sample position was adjusted to 0.1 mW. The acquisition times measured 10 s. Each spectrum made of five times was acquired.

## Supplementary Information

Supplementary Information.

## Data Availability

The data that support the findings of this study are available from Tokyo Electric Power Company Holdings Inc. but restrictions apply to the availability of these data, which were used under license for the current study, and so are not publicly available. Data are however available from the authors upon reasonable request and with permission of TEPCO HD.
