# Supplementary material for: Analysis of particles containing alpha-emitters in stagnant water at torus room of Fukushima Dai-ichi Nuclear Power Station’s Unit 2 reactor
Source: Sci Rep. 2022 May 16;12:7191. doi: 10.1038/s41598-022-11334-1 (PMC9110416; doi:10.1038/s41598-022-11334-1)
Supplement: Supplementary file 1 — Supplementary Information. [file 41598_2022_11334_MOESM1_ESM.docx]

Supplementary Information for

Analysis of particles containing alpha-emitters in stagnant water at torus room of Fukushima Dai-ichi Nuclear Power Station’s Unit 2 reactor

# T. Yomogida^1,*^, K. Ouchi^1^, T. Oka^1^, Y. Kitatsuji^1^, Y. Koma^2^ & K. Konno^3^

1Nuclear Science and Engineering Center, Japan Atomic Energy Agency, Ibaraki, 319-1195, Japan

2 Collaborative laboratories for Advanced Decommissioning Science(CLADS), Japan Atomic Energy Agency, Ibaraki 319-1194, Japan

3 Fukushima Daiichi Decontamination & Decommissioning Engineering Company, Tokyo Electric Power Company Holdings Inc., Fukushima, 979-1301, Japan,

*[Corresponding author: Takumi YOMOGIDA (yomogida.takumi@jaea.go.jp](mailto:corresponding.author@email.example))


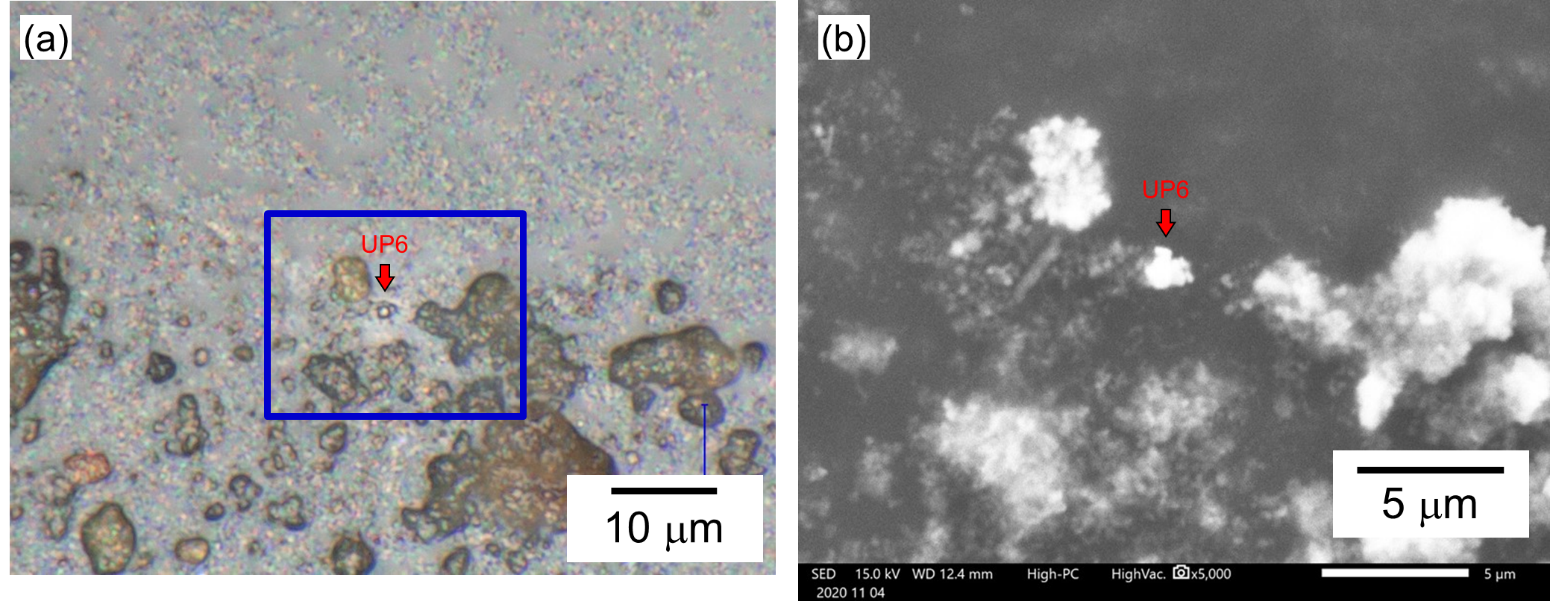


**Figure S1.** Example of the detection of U particle which was present on their own. (a) Optical image and (b) SEM image of U particle (UP6) at high magnification indicated by the blue square in (a).


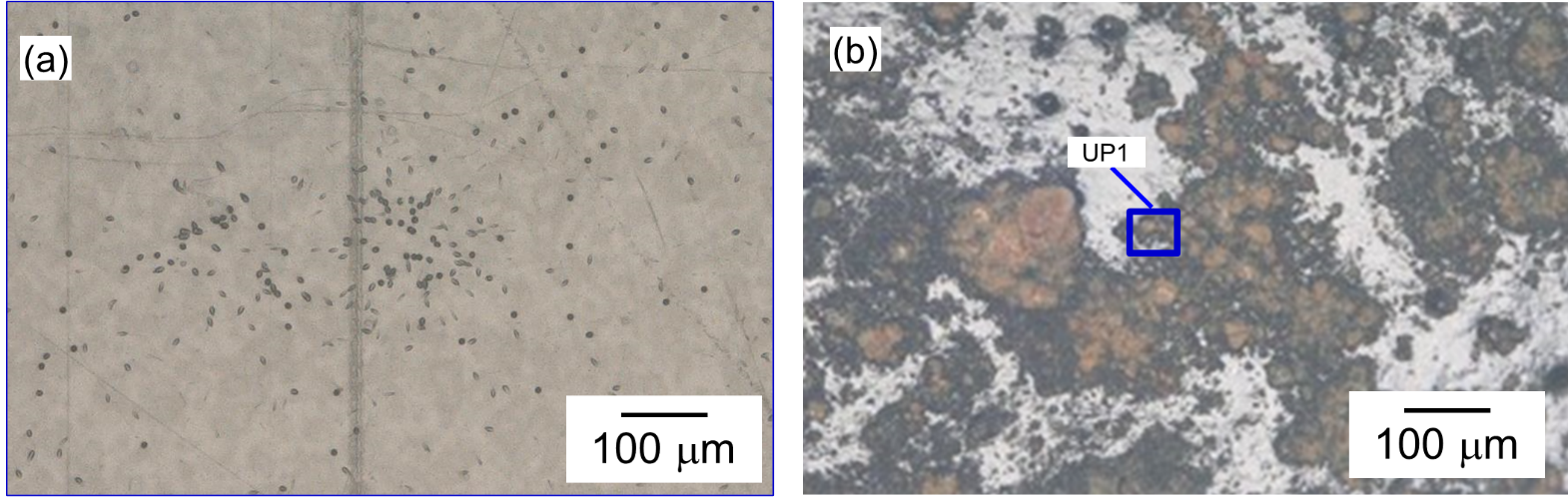


**Figure S2.** Optical images of (a) alpha tracks and (b) iron particles. UP1 indicated by blue square.

| Fraction  (μm) | Dilution rate for alpha-spectrometry | Count rate  (cps) | Concentration of all alpha particles (Bq/mL) |
| --- | --- | --- | --- |
| >10 | 76.6 | 0.1 | 86.3 |
| 1~10 | 1 | 0.01 | 0.0015±0.0004 |
| 0.1~1 | 1 | ND | <0.00093 |
| 0.02~0.1 | 1 | ND | <0.00093 |
| <0.02 | 99.5 | ND | <0.093 |
| Total | - | - | 86.3 |

**Table S1.** Radioactivity in the fraction of the contaminated water sample.

| No. | Composition [Atom%] | | | | | | | | | | | | | | | | U/(Zr+U)[%] |
| --- | --- | --- | --- | --- | --- | --- | --- | --- | --- | --- | --- | --- | --- | --- | --- | --- | --- |
|  | C | O | Na | Mg | Al | Si | S | Cl | Ca | Cr | Fe | Zn | Zr | Mo | U | SUM |  |
| UP1 | 24.77±0.23 | 50.02±0.52 | 1.00±0.06 | 0.49±0.04 | 1.35±0.05 | 0.95±0.04 | 0.37±0.02 | 0.92±0.04 | - | 0.43±0.03 | 6.39±0.14 | 0.84±0.08 | 3.96±0.06 | - | 8.52±0.10 | 100 | 68.3% |
| UP2 | - | 56.45±0.61 | 0.59±0.06 | 0.54±0.05 | 1.66±0.07 | 3.54±0.09 | 0.35±0.03 | 0.59±0.04 | - | 7.84±0.17 | 10.55±0.22 | 0.63±0.09 | 4.96±0.08 | - | 12.30±0.15 | 100 | 71.4% |
| UP3 | 25.47±0.22 | 54.68±0.46 | 1.52±0.06 | 0.38±0.03 | 1.44±0.04 | 1.04±0.04 | 1.05±0.03 | 0.70±0.03 | 0.68±0.03 | 0.11±0.02 | 5.74±0.11 | 0.39±0.05 | 2.26±0.04 | - | 4.54±0.06 | 100 | 66.8% |
| UP4 | 20.11±0.19 | 49.04±0.47 | 1.20±0.06 | 0.73±0.04 | 1.09±0.04 | 1.19±0.04 | 0.47±0.03 | 1.18±0.04 | - | 0.78±0.04 | 7.84±0.15 | 0.75±0.08 | 4.43±0.06 | - | 11.21±0.11 | 100 | 71.7% |
| UP5 | 15.20±0.21 | 38.39±0.52 | 1.08±0.08 | 0.65±0.05 | 1.98±0.08 | 2.93±0.08 | 0.94±0.04 | 2.21±0.07 | 0.32±0.03 | 0.26±0.04 | 15.97±0.27 | 1.50±0.14 | 3.44±0.07 | - | 15.14±0.17 | 100 | 81.5% |
| UP6 | - | 60.70±0.99 | - | 0.23±0.06 | 0.35±0.06 | 0.75±0.07 | - | - | - | 0.55±0.07 | 3.02±0.18 | 0.40±0.12 | 10.74±0.17 | - | 23.25±0.31 | 100 | 68.4% |
| UP7 | - | 63.12±1.76 | - | 0.85±0.16 | 2.74±0.24 | 1.85±0.19 | - | - | - | 2.70±0.27 | 11.58±0.62 | 1.18±0.32 | 4.71±0.21 | - | 11.27±0.39 | 100 | 70.5% |
| UP8 | 20.66±0.48 | 18.61±0.75 | 0.43±0.11 | - | 0.90±0.11 | 0.96±0.11 | - | 0.73±0.08 | - | - | 38.63±0.93 | 1.51±0.32 |  | 0.19±0.03 | 17.38±0.38 | 100 | 100.0% |
| UP9 | - | 62.33±1.19 | 1.81±0.18 | 0.77±0.10 | 2.36±0.15 | 1.36±0.11 | 3.09±0.14 | 0.92±0.08 | 2.18±0.13 | 1.81±0.15 | 11.30±0.41 | 0.83±0.19 | 3.16±0.11 | - | 8.08±0.22 | 100 | 71.9% |
| UP10 | 17.81±0.47 | 12.33±0.71 | - | 1.57±0.19 | 0.66±0.11 | 2.66±0.21 | 0.37±0.07 | 2.22±0.17 | - | - | 28.90±0.93 | 2.70±0.49 | 0.41±0.06 | - | 30.37±0.59 | 100 | 98.7% |
| UP11 | 9.47±0.33 | 44.50±0.95 | 1.08±0.15 | 0.80±0.10 | 2.95±0.17 | 3.70±0.17 | 1.00±0.08 | 2.36±0.12 |  | - | 22.28±0.57 | 2.16±0.29 | 0.79±0.06 | - | 8.90±0.23 | 100 | 91.8% |
| UP12 | - | 65.00±0.70 | 0.71±0.07 | 0.61±0.05 | 0.98±0.06 | 1.11±0.06 | - | 0.35±0.03 | - | 3.27±0.11 | 7.31±0.19 | 0.48±0.09 | 6.32±0.09 | - | 13.85±0.16 | 100 | 68.7% |
| UP13 | 28.39±0.27 | 45.72±0.48 | 1.62±0.08 | 5.07±0.11 | 1.19±0.05 | 4.24±0.08 | 0.42±0.02 | 1.67±0.05 | - |  | 5.26±0.13 | 0.73±0.08 |  | - | 5.69±0.08 | 100 | 100.0% |
| UP14 | 17.50±0.31 | 56.84±0.79 | 0.84±0.09 | 0.34±0.05 | 2.36±0.10 | 1.00±0.06 | - | 0.72±0.05 | - | 2.67±0.12 | 7.46±0.23 | 0.52±0.10 | 3.13±0.08 | - | 6.63±0.13 | 100 | 67.9% |

**Table S2.** Elemental composition of U particles by SEM-EDS.
